# NURSE STAFFING AND PRN PSYCHOTROPIC DRUG USE IN VA HOSPITALS

**DOI:** 10.1093/geroni/igae098.0716

**Published:** 2024-12-31

**Authors:** Ronald Shorr, Wei-Hsuan Lo-Ciganic, Bridget Hahm, Julia Gamble, Gail Powell-Cope, Stephen Luther, Seonkyeong Yang

**Affiliations:** Malcom Randall Veterans Affairs Medical Center, Gainesville, Florida, United States; Malcom Randall Veterans Affairs Medical Center, Gainesville, Florida, United States; Edward Hines, Jr. VA Hospital, Oak Park, Illinois, United States; VA Central office, Washington, District of Columbia, United States; University of California Davis, Davis, California, United States; Supervisory Health Science Specialist, Tampa, Florida, United States; University of Pittsburgh, Pittsburgh, Pennsylvania, United States

## Abstract

Reducing physical and chemical restraint use in hospitals is vital for patient safety. Studies have linked excessive physical restraint use to nurse shortages and increased risks (e.g., deaths). PRN (as needed) psychotropic drugs are used in hospitals for behavioral disturbances, yet their safety remains debatable. The impact of nurse staffing on PRN psychotropic use is unclear. Using data from 166 nursing units in Veterans Affairs (VA) hospitals from October 1, 2012, to September 30, 2017, we examined the relationship between PRN psychotropic use (i.e., antipsychotics or benzodiazepines) and two nursing staffing indicators: total nursing hours per patient day (TNHPPD) and the skill mix of registered nurses (RN), namely the proportion of nursing hours provided by RNs. Of 9,770 unit months (average 565.4 person-days of care), 25.8% involved veterans aged ≥75, 95.3% were male, and 31.6% had high Nosos risk scores. We categorized each unit into one of four quantiles based on their monthly TNHPPD, with mean and standard deviation ranging from 7.0 (1.5) in the lowest group to 15.2 (3.0) in the highest group. RN skill mix decreased as TNHPPD increased, from 71.5% to 63.1%. PRN psychotropic was 1.4%, consistent across TNHPPD levels. Multivariable mixed modeling showed PRN psychotropic use was not associated with TNHPPD levels (p=0.27) or RN skill mix (p=0.07). Our findings indicate that nurse staffing levels were not associated with PRN psychotropic use in VA hospitals.

